# Differential effects of heat-not-burn, electronic, and conventional cigarettes on endothelial glycocalyx

**DOI:** 10.1093/ehjimp/qyad008

**Published:** 2023-07-07

**Authors:** Ignatios Ikonomidis, Konstantinos Katogiannis, Kallirhoe Kourea, Gavriela Kostelli, George Pavlidis, John Thymis, Eleni Katsanaki, Eirini Maratou, Vaia Lambadiari

**Affiliations:** 2nd Cardiology Department, University Hosptal Attikon, National & Kapodistrian University of Athens, Rimini 1, Haidari, 12462 Athens, Greece; 2nd Cardiology Department, University Hosptal Attikon, National & Kapodistrian University of Athens, Rimini 1, Haidari, 12462 Athens, Greece; 2nd Cardiology Department, University Hosptal Attikon, National & Kapodistrian University of Athens, Rimini 1, Haidari, 12462 Athens, Greece; 2nd Cardiology Department, University Hosptal Attikon, National & Kapodistrian University of Athens, Rimini 1, Haidari, 12462 Athens, Greece; 2nd Cardiology Department, University Hosptal Attikon, National & Kapodistrian University of Athens, Rimini 1, Haidari, 12462 Athens, Greece; 2nd Cardiology Department, University Hosptal Attikon, National & Kapodistrian University of Athens, Rimini 1, Haidari, 12462 Athens, Greece; 2nd Cardiology Department, University Hosptal Attikon, National & Kapodistrian University of Athens, Rimini 1, Haidari, 12462 Athens, Greece; 2nd Department of Internal Medicine, Research Unit and Diabetes Centre, ‘Attikon University Hospital’, Medical School, National and Kapodistrian University of Athens, Athens, Greece; 2nd Department of Internal Medicine, Research Unit and Diabetes Centre, ‘Attikon University Hospital’, Medical School, National and Kapodistrian University of Athens, Athens, Greece

## Introduction

Heat-not-burn cigarettes (HNBC) and electronic cigarette (Ecig) constitute non-combustible smoking products. Studies report detrimental acute effects on cardiovascular function after use of these novel smoking products.^1,2^ However, the amount of inhaled chemical products, such as nitrosamines and aldehydes, after use of these products is considerably lower than that after conventional tobacco smoking (Tcig). Thus, switching from Tcig to HNBC or Ecig may improve cardiovascular function in the long term.^1,2^ However, the effect of HNBC and Ecig use on endothelial glycocalyx has not been investigated so far.

## Methods

Out of 100 screened smokers attending our smoking cessation unit, (i) 50 current smokers were randomized to HNBC use (*n* = 25) or to Ecig puffing (*n* = 25) and (ii) 50 smokers, who were seeking for information about smoking cessation programmes but were unwilling to participate within the next month, were used as controls. The controls were matched with HNBC and Ecig users for age, sex, and pack-years. Smoking status was verified by self-reported smoking burden per day and primarily exhaled carbon monoxide (eCO) concentration measurement [parts per million (ppm), Bedfont Scientific, Maidstone, Kent, UK]. Exclusion criteria were smoking less than five cigarettes per day end eCO < 10 ppm, history of cardiovascular or other systemic disease, and use of any medication.

Smokers used either a mainstream Tcig [Marlboro Red, Papastratos-Philip Morris International (PMI), Athens, Greece] or a HEETS stick (PMI, amber flavour) with a commercially available HNBC (IQOS, PMI). The Ecig that was used contained liquid with nicotine concentration of 12 mg/mL (NOBACCO eGo Epsilon BDC 1100, eGo battery, 1100 mAh, operating at 3.9 V—propylene glycol 74.3%, glycerin 20%, flavouring 4.5%, and nicotine 1.2%/12 mg/mL).

**Figure 1:**
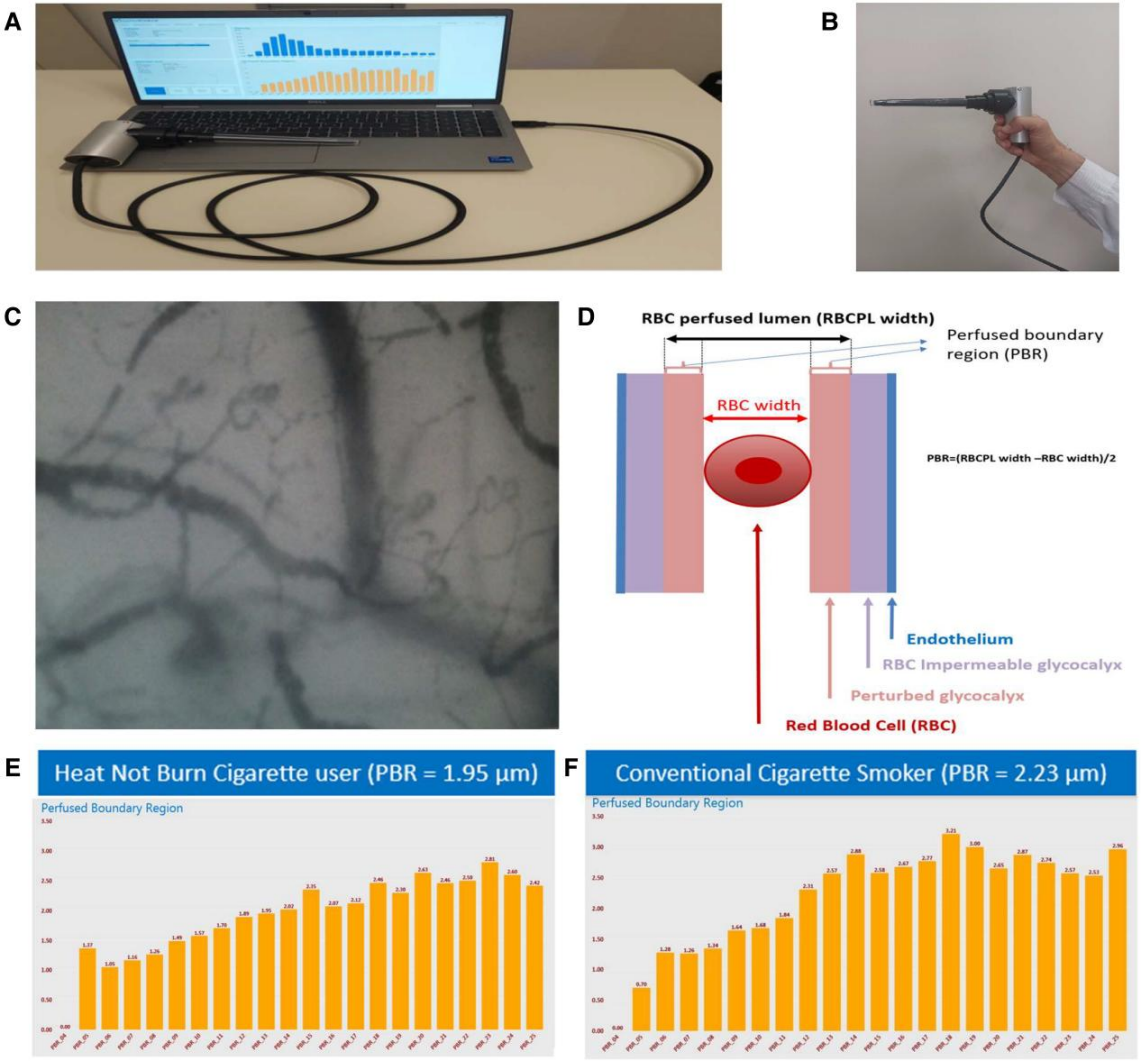
Assessment of perfused boundary region of the sublingual arterial micro-vessels, by GlycoCheck software. RBCs, red blood cells; SDF, sideview darkfield imaging; PBR, perfused boundary region. (*A*) SDF camera and GlycoCheck software. GlycoCheck software automatically analyses the recordings and then estimates the PBR for each vascular increment diameter. (*B*) SDF camera utilizes green reflected light-emitting diode (LED) light (540 nm) from haemoglobin molecules to depict the radial displacements of RBCs within the micro-vessels. The camera is inserted under the tongue and captures more than 3000 vascular segments of vessels with diameter ranging from 5 to 25 *μ*m. (*C*) Example of recording of sublingual micro-vessels by SDF camera. The camera captures more than 3000 vascular segments of vessels. (*D*) Schematic illustration of a micro-vessel, displaying the perfused boundary region of arterial micro-vessel. PBR is calculated by the formula (RBC perfused lumen width—RBC width)/2. (*E*) Each column illustrates micro-vessels’ diameters. PBR within normal range for micro-vessels with diameter 5–25 *μ*m (mean value of PBR5-25 = 1.95 *μ*m) indicating intact glycocalyx layer of a subject randomized to use HNBC. (*F*) Each column illustrates micro-vessels’ diameters. Increased PBR (mean value of PBR5-25 = 2.23 *μ*m) indicating impaired glycocalyx of a subject smoking conventional cigarette, perturbed by red blood cells. The bars on image *F* are higher than the respective bars on image *E*.

GlycoCheck (Microvascular Health Solutions Inc., Salt Lake City, UT, USA) is a device that performs sublingual microscopy by a dedicated camera (sidestream darkfield imaging) and measures capillaries diameters between 5 and 25 *µ*m, and, most importantly, the perfused boundary regions (PBR) in straight sections of these vessels. The PBR mirrors the glycocalyx thickness, as it is a measure for the deviation of red blood cells from the optimal middle line movement within the damaged endothelial glycocalyx layer (optimal if the negatively charged glycocalyx on both erythrocytes and endothelial cells is intact). The higher the PBR the more damaged the glycocalyx thickness (*Figure 1*).

eCO, endothelial glycocalyx, and blood cotinine were assessed at baseline and after 1 month by a single operator, blinded to intervention and to values of measured biomarkers. Participants were instructed to abstain from smoking in the morning before the evaluation. Adherence was assessed by the daily use of HNBC sticks or Ecig liquid and by CO measurement at Days 15 and 30.

## Results

The Tcig smokers were of age 48 ± 5 years, 53% female, and used 27 ± 9 cigarettes/day, 29 ± 9 pack-years; the HNBC users were of age 46 ± 14 years, 55% female, and used 26 ± 8 cigarettes/day, 30 ± 8 pack-years; the Ecig users were of age 45 ± 8 years, 51% female, and used 27 ± 10 cigarettes/day, 28 ± 10 pack-years.

Compared with baseline, switching to HNBC for 1 month improved only PBR_20–25_ (2.55 ± 0.49 vs 2.34 ± 0.34 *μ*m, *P* = 0.002). Puffing Ecig did not alter endothelial glycocalyx at any micro-vessel range throughout the study (*P* > 0.05). Conversely, smoking Tcig for 1 month further deteriorated endothelial glycocalyx at all micro-vessel ranges compared with baseline (*Table 1*, *P* < 0.05).

**Table 1:** Effects of heat-no-burn, electronic, and conventional cigarettes on endothelial glycocalyx, carbon monoxide, and inhaled nicotine

	Group	Baseline	1 month	*P*-value
PBR (5–25 *μ*m)	HNBC	2.21 ± 0.27	2.23 ± 0.28	0.732
	Ecig	2.13 ± 0.25	2.12 ± 0.19	0.899
	Tcig	2.18 ± 0.24	2.28 ± 0.19	0.022
PBR (5–9 *μ*m)	HNBC	1.20 ± 0.14	1.20 ± 0.21	0.979
	Ecig	1.18 ± 0.13	1.12 ± 0.16	0.281
	Tcig	1.15 ± 0.10	1.25 ± 0.14	0.001
PBR (10–19 *μ*m)	HNBC	2.27 ± 0.26	2.35 ± 0.34	0.279
	Ecig	2.28 ± 0.28	2.27 ± 0.25	0.959
	Tcig	2.21 ± 0.21	2.41 ± 0.18	<0.001
PBR (20–25 *μ*m)	HNBC	2.55 ± 0.49	2.34 ± 0.34	0.002
	Ecig	2.65 ± 0.48	2.68 ± 0.34	0.834
	Tcig	2.59 ± 0.43	2.79 ± 0.36	0.029
CO (ppm)	HNBC	13.9 ± 5.4	6.2 ± 6.4	<0.001
	Ecig	13.5 ± 4.4	5.6 ± 4.7	<0.001
	Tcig	14.8 ± 4.9	16.1 ± 4.8	0.312
Cotinine (ng/mL)	HNBC	90.34 ± 30.24	93.18 ± 28.31	0.432
	Ecig	90.34 ± 30.24	91.18 ± 27.31	0.535
	Tcig	88.13 ± 31.87	93.18 ± 30.42	0.489

Parameters are expressed as mean values ± standard deviation (SD)

Ecig, electronic cigarette; HNBC, heat-not-burn cigarette; Tcig, tobacco (conventional) cigarette.

Cotinine blood levels were similar between baseline and after 1 month using HNBC (*P* = 0.432), Ecig (*P* = 0.535), or Tcig (*P* = 0.489).

Compared with baseline, CO was decreased in HNBC and Ecig users (mean per cent change: −55% and −58%, respectively *P* < 0.001), while remained unchanged in Tcig smokers (*P* = 0.312) at 1 month. Thus, Tcig smokers had higher CO than those who switched to HNBC or Ecig at 1 month (*P* < 0.01).

## Conclusions

In this study, we observed that conventional cigarette smokers further deteriorated endothelial glycocalyx after continuation of smoking for 1 month. Conversely, smokers switching to Ecig preserved glycocalyx integrity while those who switched to HNBC showed a modest improvement of the glycocalyx integrity in the micro-vessel ranging from 20 to 25 *μ*m. Cotinine levels, which reflect nicotine exposure, were similar between Tcig, HNBC, and Ecig groups. Thus, impairment of endothelial glycocalyx in the Tcig group was independent from nicotine consumption and was likely related with greater exposure to toxic emissions, such as CO, after Tcig use than after switching to HNBC or Ecig.

## Lead author biography

Dr Ignatios Ikonomidis, MD, PhD, is Professor of Cardiology, Director of Echocardiography, Laboratory of Preventive Cardiology and Clinic of Cardiometabolic Diseases, 2nd Cardiology Department, National and Kapodistrian University of Athens (NKUA), Attikon Hospital, Athens, Greece. In 1997, he completed his training in Cardiology and Cardiac Imaging, in Hammersmith Hospital, Imperial College, London, UK. Since then, he worked as consultant cardiologist in University Hospital of Patras, Department of Clinical Therapeutics and 2nd Cardiology Department, Medical School, Athens. His research fields include cardio-vascular imaging, microcirculation, inflammation, diabetes, smoking control, and ventricular–arterial coupling. He authored 300 publications in indexed journals and several book chapters.
